# Natural Bovine Coronavirus Infection in a Calf Persistently Infected with Bovine Viral Diarrhea Virus: Viral Shedding, Immunological Features and S Gene Variations

**DOI:** 10.3390/ani11123350

**Published:** 2021-11-23

**Authors:** Annamaria Pratelli, Maria Stella Lucente, Marco Cordisco, Stefano Ciccarelli, Roberta Di Fonte, Alessio Sposato, Viviana Mari, Paolo Capozza, Francesco Pellegrini, Grazia Carelli, Amalia Azzariti, Canio Buonavoglia

**Affiliations:** 1 Department of Veterinary Medicine, University Aldo Moro of Bari, Strada per Casamassima km 3, 70010 Valenzano (Ba), Italy; mariastella.lucente@uniba.it (M.S.L.); marco.cordisco@uniba.it (M.C.); stefano.ciccarelli@uniba.it (S.C.); alessio.sposato@uniba.it (A.S.); viviana.mari@uniba.it (V.M.); paolo.capozza@uniba.it (P.C.); francesco.pellegrini@uniba.it (F.P.); grazia.carelli@uniba.it (G.C.); canio.buonavoglia@uniba.it (C.B.); 2 Laboratory of Experimental Pharmacology at IRCCS Istituto Tumori Giovanni Paolo II, Viale Orazio Flacco 65, 70124 Bari, Italy; difonte.roberta@gmail.com (R.D.F.); a.azzariti@oncologico.bari.it (A.A.)

**Keywords:** cattle, coronavirus, BVDV, natural infection

## Abstract

**Simple Summary:**

The evolution of a bovine coronavirus (BCoV) natural infection in a calf persistently infected with bovine viral diarrhea virus (BVDV) was described. The infected calf developed intermittent nasal discharge, diarrhea and hyperthermia. The total number of leukocytes/mL and the absolute differential number of neutrophils and lymphocytes resulted within the normal range, but the monocytes increased at T28 (time 28 post-infection) and the CD8^+^ subpopulation increased at T7 and between T28 and T35. BCoV shedding in nasal discharges and feces was detected up to three weeks post infection (p.i.) and high antibody titers persisted for up to 8 weeks p.i. Virus shedding increased until T14, contrary to what was observed in a previous study where BCoV was detected with a lower load in the co-infected (BCoV/BVDV) calves than in the calves infected with BCoV only. We can suppose that BVDV may have exacerbated the long viral excretion, as well as favoring the onset of mutations in the genome of BCoV. An extensive study was performed to verify if the selective pressure in the S gene could be a natural mode of variation of BCoV.

**Abstract:**

The evolution of a bovine coronavirus (BCoV) natural infection in a calf persistently infected with bovine viral diarrhea virus (BVDV) was described. The infected calf developed intermittent nasal discharge, diarrhea and hyperthermia. The total number of leukocytes/mL and the absolute differential number of neutrophils and lymphocytes resulted within the normal range, but monocytes increased at T28 (time 28 post-infection). Flow-cytometry analysis evidenced that the CD8^+^ subpopulation increased at T7 and between T28 and T35. BCoV shedding in nasal discharges and feces was detected up to three weeks post infection and high antibody titers persisted up to T56. The RNA BCoV load increased until T14, contrary to what was observed in a previous study where the fecal excretion of BCoV was significantly lower in the co-infected (BCoV/BVDV) calves than in the calves infected with BCoV only. We can suppose that BVDV may have modulated the BCoV infection exacerbating the long viral excretion, as well as favoring the onset of mutations in the genome of BCoV detected in fecal samples at T21. An extensive study was performed to verify if the selective pressure in the S gene could be a natural mode of variation of BCoV, providing data for the identification of new epidemic strains, genotypes or recombinant *betacoronaviruses*.

## 1. Introduction

Coronaviruses (CoVs) are enveloped viruses with large and complex RNA genomes. up to 32 kb that encode for 16 non-structural proteins regulating RNA synthesis and modifications. The *Coronaviridae* family includes four genera, *Alphacoronavirus*, *Betacoronavirus*, *Gammacoronavirus* and *Deltacoronavirus*, characterized by a variable tissue tropism and by the ability to cross interspecies barriers and to infect both mammalian and avian species, causing diseases with remarkably differences [1]. This skill is the expression of the low fidelity of the viral RNA polymerase and of the high frequency of recombination events during RNA replication, as observed in several *Alphacoronaviruses* that generate viable quasispecies pools [2,3,4,5,6].

Bovine coronavirus (BCoV) belongs to the *Betacoronavirus* genus, subgenus *Embecovirus*, and is responsible for different clinical diseases in ruminants affecting both respiratory and enteric tracts, such as neonatal diarrhea, the wintery disease in adults and respiratory diseases as a cofactor of the Bovine Respiratory Syndrome (BRD) [7,8,9,10]. Regardless of the clinical signs, BCoV, which is also commonly identified in healthy cattle [11], is shed both in the feces and in the nasal secretions. Although enteric and respiratory strains show genetic differences in the spike gene, a single serotype was identified and a cross protection among isolates was observed. Numerous investigators have confirmed that enteric and respiratory BCoVs are members of the same *quasispecies*, suggesting that different clinical signs are the expression of complex interactions among the virus, the host and the environment [9,12].

Despite the fact that BCoV infection is widespread among cattle worldwide, resulting in huge economic losses for the livestock industry, there are few reports describing the characteristic of the respiratory and enteric infections and the innate and adaptive immune responses [13,14,15,16]. Considering the extreme variability of the CoVs and the influence of the immune system on disease evolution and on the emergence of new strains, a persistent bovine viral diarrhea virus (BVDV) infection can be considered as an expression of the impairment of the immune system in order to evaluate the variation of the BCoV infection both virologically and immunologically. BVDV may cause transient and persistent infections, the latter characterized by immunotolerance specific for the infecting viral strain. Transient and persistent infections differ substantially in the host’s antiviral immune response, but both are associated with an increased frequency of secondary infections [17]. In vitro studies have demonstrated that non-cytopathic (ncp) BVDV fails to induce interferon type I in bovine macrophages, thus disposing a mechanism that is able to suppress a key element of the antiviral defense of the innate immune system. Moreover, since interferon is important in the activation of the adaptive immune response, the suppression of this signal may be essential for the establishment of persistent infection and immunotolerance [17]. Several studies were performed for the evaluation of the adaptive immunity in the persistent infected (PI) animals [18,19]. Chase [20] observed that T-lymphocytes numbers are reduced following BVDV infection in a strain dependent manner, defining that ncp BVDV may dispose a mechanism that is able to suppress a key element of the antiviral defense of the innate immune system. The present report describes the virological and immunological variation of a natural BCoV infection in a calf persistently infected with BVDV. The respiratory and the enteric shedding of BCoV, the innate and the adaptive immune response to BCoV and the variation of the spike protein during infection were evaluated.

## 2. Materials and Methods

### 2.1. Clinical Case

In the first week of April 2021, one 11-month-old female PI calf (Brown breed) was identified in a herd of Apulia region, Italy, that consisted of 99 Alpine Brown cattle, all under the age of 1 year, including 91 lactating cows. The herd adheres to the compulsory eradication plans for tuberculosis, brucellosis and bovine leucosis and to the voluntary eradication plan for BVDV. The PI animals are destined to be slaughtered. The PI calf was taken to the Veterinary Hospital of the Department of Veterinary Medicine of the University of Bari, Italy, for a diagnostic confirmation of BVDV PI and for clinical and hematological assessments to be shown to students of the Veterinary Medicine degree course. A week before arrival, the calf was clinically examined on the farm of origin and subjected to virological and bacteriological investigations. To this purpose, nasal swabs (NSs) collected from both nasal cavities with a dry sterile swab, a fecal swab (FS) collected directly from the rectum with a similar dry sterile swab and EDTA-treated blood and serum samples were collected to monitor the health of the calf and the concomitance of other viral and/or bacterial infections. In particular, NS, FS and EDTA-treated blood were tested in RT-qPCR for BCoV, BVDV, *bovine respiratory syncytial virus* (BRSV), *bovine parainfluenza virus* (BPiV), *bovine adenovirus* (BAdV), *bovine herpesvirus type 1* (BoHV-1), *Mannheimia haemolytica*, *Pasteurella multocida*, *Histophilus somni* and *Mycoplasma bovis*. The serum sample was tested for antibodies detection using the ELISA test (Svanovir^®^ BVDV-Ab, Boehringer Ingelheim Svanova, Uppsala, Sweden) and the neutralization test for BVDV and BoHV-1, respectively. The collected samples were immediately transported on ice to the laboratory of Infectious Diseases of the Department of Veterinary Medicine of Bari (Italy) and stored at −80 °C (NS, FS) and at RT (blood samples) before processing.

The calf, which arrived on 12 April 2021 (T0), was immediately hospitalized in the Infection Diseases Unit of the Veterinary Hospital in a separate room and subjected to clinical examinations on the day of arrival and then two times a day during the entire period of hospitalization (Italian Ministry of Health authorization n. 0017484-DGSAF-MDS-P). To minimize the stress and discomfort, the calf was kept in a pen with straw bedding, was fed a commercial concentrate twice daily and had access to haylage and water ad libitum. The facility was closed for other animals and had restricted admission for people. A trained animal technician and a veterinarian monitored the calf at least two times a day. To enter the animal room area, the investigators undressed and put on work clothes and boots. The presence of the following clinical signs was noted down: rectal temperature, pain and depression, nasal discharge, polypnea, coughing and diarrhea. Two days after arrival (T1), the calf showed hyperthermia (39.8 °C) and, consequently, NS, FS, EDTA-treated blood and serum samples were collected to monitor its health status and to assess the presence of concomitant virological/bacteriological infections. The presence of BVDV was confirmed in the blood, NS and FS, and BCoV was contextually detected in the NS. As a consequence, stool samples, NSs, serum samples and EDTA-treated blood were collected weekly up to 60 days (T60) until BCoV test were negative.

### 2.2. Clinical Score and Treatment Procedures

The degrees of sensory dejection, the body temperature, the diarrhea, the cough and the nasal discharge were converted to numeric values. The values ranged from 1 to 3, corresponding to a mild, moderate and severe degree. Similarly, numerical values 1, 2 and 3 were assigned to body temperatures of 39.0–39.5, 39.6–40.0 and >40.1 °C, respectively. Individual scores for general condition, body temperature, diarrhea and respiratory tract involvement were calculated. A score above or equal to two on three consecutive days was categorized as mild clinical disease; a score above or equal to six on three consecutive days as moderate disease and a score above or equal to eleven was categorized as severe clinical disease (Table 1) [14].

Indications for antibiotic treatment (30,000 IU procaine benzyl penicillin/kg bodyweight/day i.m. for five consecutive days) were abnormal sounds on lung auscultation or prolonged high temperature, and in case of severe depression, discomfort, pain and dehydration, a non-steroidal anti-inflammatory drug (Metacam^®^ 20 mg, Boehringer Ingelheim Italia S.p.A., Milan, Italy) and an oral fluid with electrolytes were administered.

### 2.3. RT-qPCR for Virological and Bacteriological Analysis

NSs and FSs were dissolved in 1 mL of Dulbecco Minimal Essential Medium (DMEM) and centrifuged at 4000× *g* for 20 min at 4 °C. RNA was extracted from 200 µL of supernatant of each swab and from 100 µL of each EDTA-treated blood samples by using the commercial QIAamp^®^ cador^®^ Pathogen Mini Kit (Qiagen GmbH, Hilden, Germany), according to the manufacturer’s instructions, with an elution volume of 100 µL. Extracted samples were immediately stored at −80 °C until tested using RT-qPCR. The reverse transcription using random hexamers and MuLV reverse transcriptase (GeneAmp^®^ RNA PCR, Applied Biosystems, Applera Italia, Monza, Italy) was performed in a total volume of 10 μL for the detection of BCoV, BVDV, BRSV and BPiV according to the manufacturer’s protocol. Then, the RT-qPCR for the detection of the above RNA viruses and of the DNA pathogens BAdV, BoHV-1, *M. haemolytica*, *P. multocida*, *H. somni* and *M bovis* was carried out using primers and TaqMan probe and the same reaction conditions and reaction mix components, as previously reported [7,8,10]. Briefly, 10 μL of cDNA or extracted DNA were added to 15 μL of the reaction master mix (IQ™ Supermix, Bio-Rad Laboratories Srl, Segrate, Italy) containing 0.6 μM of each primer and 0.4 μM probe. After the activation of iTaq DNA polymerase at 95 °C for 10 min, the thermal cycling for amplification consisted of 45 cycles of denaturation at 95 °C for 10 s and an annealing extension at 56 °C for 30 s. RT-qPCR was performed in an i-Cycler iQTM Real-Time Detection System (Bio-Rad Laboratories Srl) and the data were analyzed with the proper sequence detector software (version 3.0).

### 2.4. Virus Detection

RT-qPCR positive samples were also cultured for the detection of BCoV. The NS and FS supernatants, prepared as a 10% suspension in DMEM and containing 5000 IU/mL penicillin, 2500 µg/mL streptomycin, 10 µg/mL amphotericin and 1 μg/mL trypsin, were used for virus isolation. Similarly, each positive EDTA-treated blood sample was treated as described. A monolayer of 2-days-old Human Rectal Tumor (HRT)-18 cells in a 24-well plate were infected, then incubated at 37 °C in a 5% CO_2_ incubator and observed daily for cytopathic effects (cpe). After 4 days of incubation, in the absence of cpe, infected cells were frozen and thawed 3 times and 2 additional passages were performed. Indirect immunofluorescence (IF) tests with specific antisera against BCoV were also performed on the infected cells after 2-day intervals.

### 2.5. Blood Count and Peripheral Blood Mononuclear Cell (PBMC) Isolation Characterization Using Flow Cytometry

The blood count was performed on the whole blood sample collected in EDTA tubes, using Cell Dyne 3700 (Abbot). The number of total leukocytes/mL and the absolute differential number of neutrophils, lymphocytes and monocytes were examined.

An aliquot of the EDTA blood samples was treated for PBMC characterization using flow cytometry. PBMCs were isolated from 3 mL of peripheral blood using Ficoll–Hypaque gradient centrifugation. The separated cells, diluted in phosphate-buffered saline (PBS) in a 1:1 ratio, were carefully layered on top of the Ficoll-Hypaque and then processed as previously described [21]. The isolated PBMCs were labelled with anti-bovine antibodies CD4-FITC (clone CC8), CD8-PE (clone CC63), CD14-FITC (clone TüK4) and CD335-NKp46-PE (clone AKS1) (Thermo Fischer, UK) for 30 min at 2–8 °C in the dark, according to the manufacturer’s instructions. After staining, the labelled PBMCs, washed with PBS, were collected and then analyzed using an Attune TMNxT Acoustic Focusing Cytometer (ThermoFisher, Waltham, MA, USA). For sample reading, the Cytometer was equipped with four lasers (405 nm (violet), 488 nm (blue), 561 nm (yellow) and 637 nm (red)). The data were analyzed using AttuneTM NxT Analysis Software (ThermoFisher) [21].

### 2.6. Serological Tests

Serial two-fold dilutions of serum samples (starting from 1:10) were tested for antibodies to BCoV using the immunofluorescence (IF) and the seroneutralization (SN) test. IgAs antibodies detection in the nasal secretions (serial two-fold dilutions from 1:5) was carried out with the IF test. The IF test was performed using *Madin Darby Bovine Kidney* (MDBK) cells infected with an in vitro-adapted BCoV strain 16/20, deposited on multispot slides and acetone-fixed. The anti-bovine IgG fluorescein conjugated serum (Sigma Chemicals, St. Louis, MO, USA) and the anti-bovine IgA fluorescein conjugated serum (Sigma Chemicals, St. Louis, MO, USA) were employed for serum and mucosal antibodies, respectively.

SN was carried out in 96-well microtiter plates containing a 24-h monolayer of MDBK cells. Serum dilutions were mixed with 100 Tissue Culture Infectious Doses (TCID)_50_ of BCoV strain 16/20 and after a 1-h incubation at room temperature, were added to each well. After five days of incubation at 37 °C in a CO_2_ incubator, the plates were read with an IF test using an inverted fluorescence microscope. The titer was expressed as the highest serum dilution neutralizing the virus (absence of fluorescence).

### 2.7. Sequence Analysis of BCoV S Protein

BCoV RNA, extracted with the commercial IndiSpin^®^ Pathogen Kit (Indical Bioscience) from NSs collected at T7, T14 and T21 and from FSs collected at T21, TR22 and TR23 according to manufacturer’s instructions, was subjected to amplification of the S gene, using SuperScript™ One-Step RT-PCR System with Platinum™ Taq DNA Polymerase (Thermo Fisher-Invitrogen). Six overlapping fragments were amplified using six primer pairs 9F (TATGATCCGCTACCAATTATTTTGCTTGGCA)/9R(ACAACACCAGTGTCTGTAAAATATGCA), 15F(TTCCTGTTTATATAAGCGTAATTTC)/15R(CACCTATCCCCTTGTAAACAAGAGTC), 1F(ACTTAGTTGGCATAGGTGAGCACTGTTC)/1R(ACATGCTACATAATCACCACAGACAA), S2417F(TTACTATAGGTAACATGGAGGAGTTTA)/S3330R(AGTGTAGAGTCACTAAGCTGTTGAGAA), 16F(TAATGCAAATGCTGAAGCTCTTAATAAC)/16R(TGTGATGTTTTAATTACTAACTCCTGGTGTCC), 4F(GGTGGTTGTTGTGATGATTATACTGGACA)/4R(ACTACAACTATTATAACCAATAAACAAAT).

The PCR products were sequenced by BaseClear BV (Leiden-The Netherland) and analyzed using Geneious Prime software version 2019.0.4 (Biomatters Ltd., Auckland, New Zealand), the NCBI’s (http://www.ncbi.nlm.nih.gov (accessed on 15 June 2021)) and EMBL’s (http://www.ebi.ac.uk (accessed on 15 June 2021)) analysis tools.

Multiple-sequence alignments with reference *Betacoronavirus* strains (BCoV/HCoV/CRCoV/BuCoV) were constructed by using Clustal W (http://www.ebi.ac.uk/clustalw (accessed on 15 June 2021)) (GeneBank accession numbers EU14648.1, EF445634.1, DQ915164.2, DQ811784.2, AF391541.1, KX344031.1, AF058943.1, U00735.2, AF220295, EU19216.1, EU999954, KX432213.1).

## 3. Results

All the samples collected in the farm of origin a week before the arrival at the Department of Veterinary Medicine were tested in RT-qPCR and the results were negative, except for the BVDV identified in the NS, FS and in the blood. The serum sample tested negative for BVDV and BoHV-1 antibodies.

**Figure 1:**
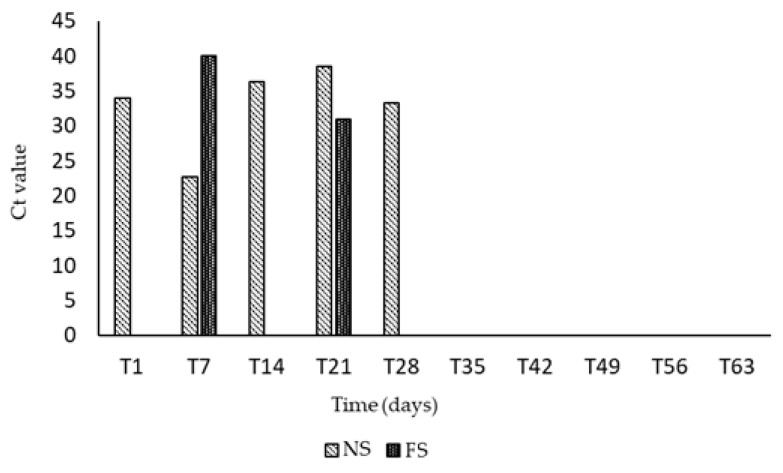
Weekly virological monitoring of BCoV RNA detected in NSs and FSs. BCoV RNA was revealed intermittently from T1 to T28 in the nasal secretions, and from T7 to T21 in the feces.

On 12 April 2021, the day of arrival at the Veterinary Hospital of the Department of Infectious Diseases (T0), the calf was clinically examined, and no relevant clinical features were observed. Two days later (T1), the calf showed hyperthermia (39.8 °C) and BCoV was detected in the NSs. The weekly virological monitoring pointed out that the PI calf was co-infected with BCoV, and viral RNA was excreted in the NSs starting from T1 for up to 14 days (T14). Intermittent and moderate excretion continued up to T28. Nasal shedding consistently preceded fecal excretion and BCoV RNA was intermittently detected in the FSs from T7 to T21, with the highest excretion at T21. Interestingly, after the first day of excretion (T7), fecal shedding was not detected at T14, but it restarted at T21. BCoV RNA was no longer identified in both NSs and FSs from T35 and it was never detected in any blood samples tested (Figure 1).

The NSs and the FSs positive in the RT-qPCR were cultured on HRT-18 cells in a 24-well plate for BCoV isolation. Only one sample, a NS collected at T7, had a positive result by IF test performed 4 days post infection at the first cell cultured passage. No cpe was detected in any of the two additional passages performed.

**Figure 2:**
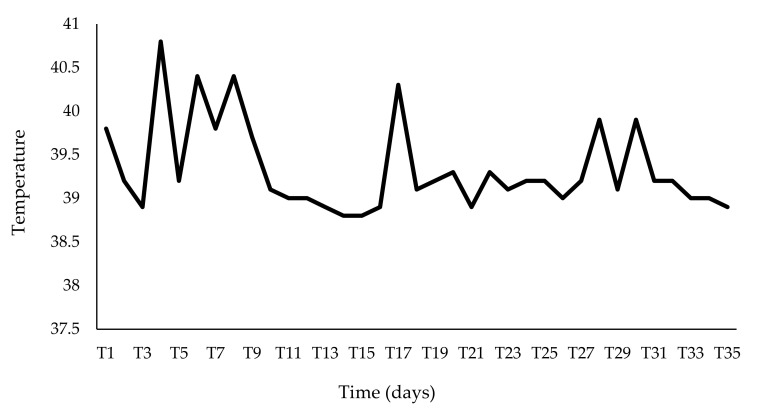
Trend of the temperature detected during the observation period. The fever compared two days after the arrival (T1) and lasted for almost the entire period of viral excretion, with fever peaks observed at T4, T6, T8, T17.

The calf was clinically monitored two times a day by a veterinarian. Hyperthermia, which appeared two days after the arrival (T1), lasted for almost the entire period of viral excretion (Figure 2).

**Figure 3:**
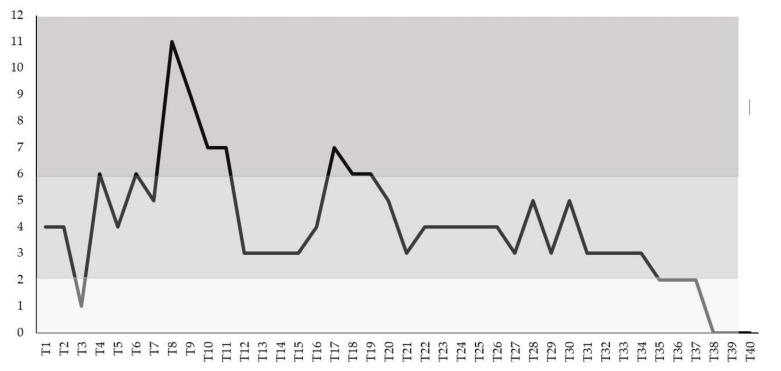
Clinical score in the BCoV naturally infected calf calculated based on daily registrations of clinical signs. A score above or equal to six on three consecutive days was observed from T8 to T11 and from T17 to T19 (moderate disease), while a score of eleven was revealed only at T8.

The calf developed signs of respiratory disease, including pyrexia up to 40.8 °C (T4), moderate serous-mucous nasal discharge from T8 to T11, with spot at T18 and T19, a spontaneous dry cough of variable severity from T1 to T11 and from T18 to T34 and moderate intermittent diarrhea from T8 to T26. Depression was constantly observed until T30, and the calf showed anorexia during the first three weeks, mostly coinciding with the fever peaks (T4, T6, T8, T17) and these were not considered prominent clinical features. A score above or equal to six on three consecutive days was observed from T8 to T11 and from T17 to T19 (moderate disease), while a score of eleven was revealed only at T8 when the calf showed marked pyrexia and moderate depression and diarrhea (Figure 3). The cattle recovered without antibiotic treatment.

The severity of the clinical signs was compared with RT-qPCR results. A correlation was observed between the overall clinical score and the presence of BCoV in the collected samples, and mostly the best association was observed when viral RNA detection was compared to hyperthermia and sensory depression (Table 2).

The blood samples, FSs and NSs collected weekly and contextually tested for BVDV had steadily positive results and, compared to T1, an increase in the amount of RNA in the blood (Ct + 3.34), in the FSs (Ct + 3.2) and mostly in the NSs (Ct + 6.46) was observed during the first two weeks of observation.

**Figure 4:**
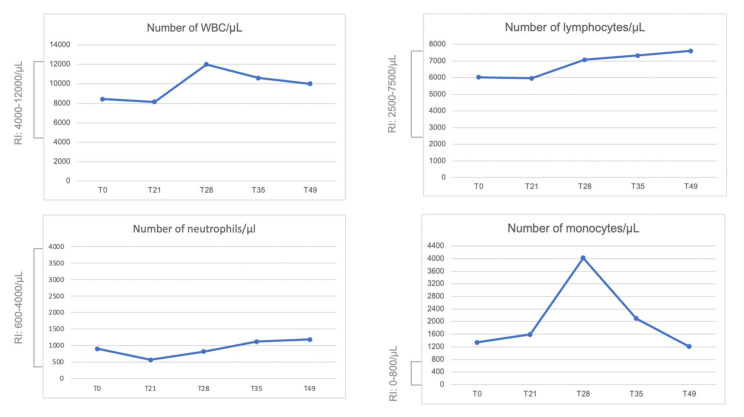
Blood count. Total white blood cells (WBCs), absolute differential number of lymphocytes, neutrophils and monocytes per microliter (µL) observed during the monitoring. RI: Reference Interval.

The total number of leukocytes/μL and the absolute differential number of neutrophils, lymphocytes and monocytes are reported in Figure 4. No relevant variation was observed except for the monocytosis observed at T28, but the number of monocytes was above the range at T0.

**Figure 5:**
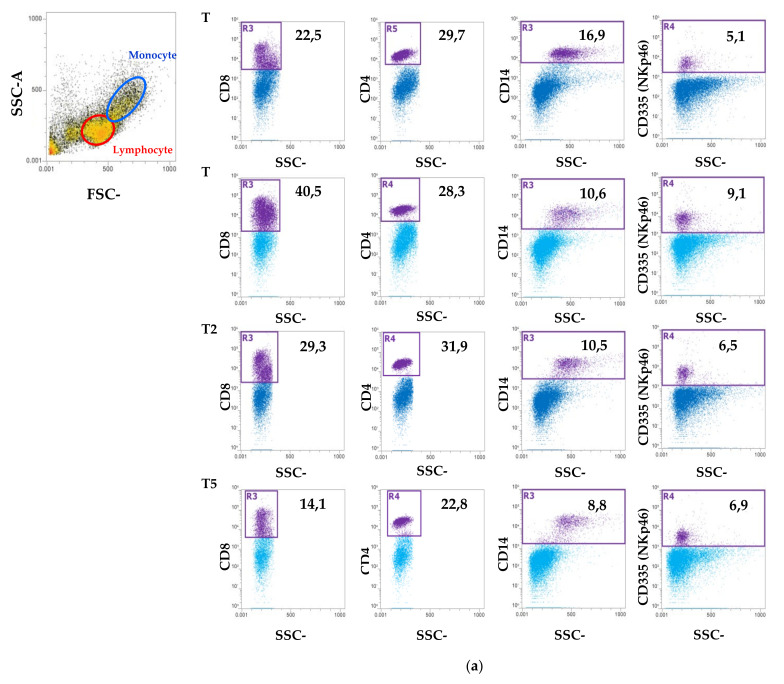

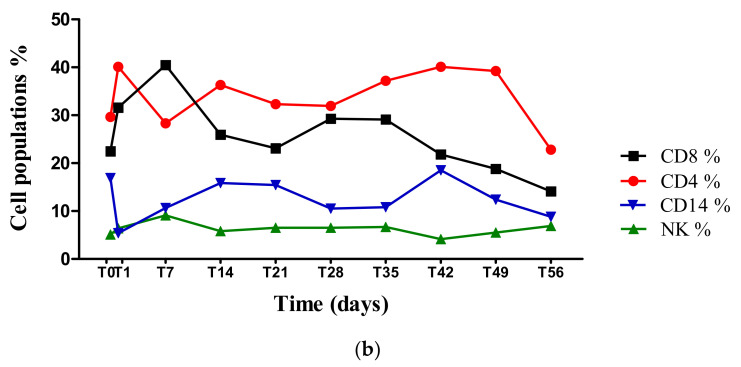
Flow cytometric analysis of immune cells population in PBMCs. (**a**) Representative dot plots showing the selection of lymphocytes and monocytes based on their SSC/FSC properties and the percentage of CD4^+^, CD8^+^, CD14^+^ and CD335^+^ (NK) cells at T0 (the day of the arrival at the Department of Veterinary Medicine), T1, T28 and T56. (**b**) Line plot that shows the trend of the CD4^+^, CD8^+^, CD14^+^ and CD335^+^ (NK) cell populations (%) at different time points from T0 to T56.

In order to determine blood cells population variations and the dynamics of the lymphocytes and the monocytes during BCoV infection, flow cytometry analysis was performed and the PBMCs collected weekly from T0 to T56 were characterized by the surface staining of CD4, CD8, CD14 and CD335. Lymphocyte and monocytes were identified by light scatter properties and further subgated for the expression of CD8, CD4 and CD335 (NK) and for CD14, respectively. Flow cytometry analysis evidenced that the CD8^+^ subpopulation showed an increase at T7, and the same subpopulation showed a second increase between T28 and T35. All the other PBMC populations showed no further modulations (Figure 5).

**Figure 6:**
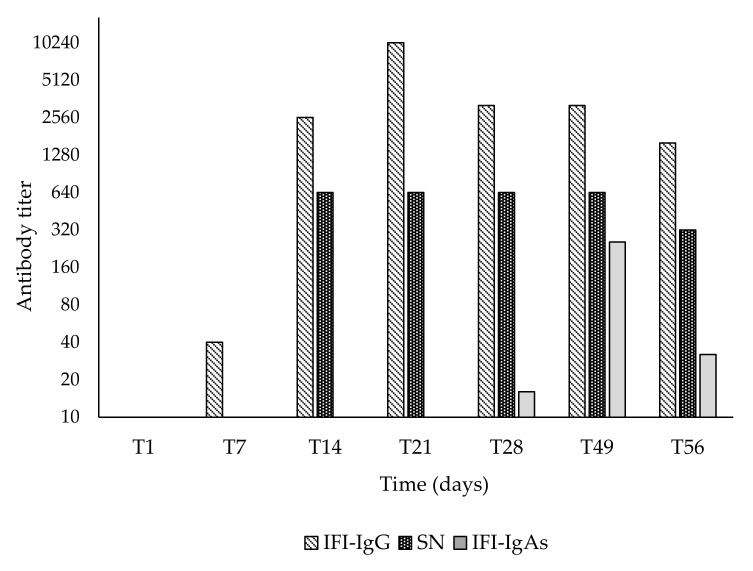
Antibodies to BCoV. Serum IgG and nasal IgAs antibodies detected with IF assay and serum antibodies detected with SN test.

The serum BCoV IgG antibodies were measured weekly and with an IF test, moderate seroconversion was already observed at T7. The antibodies titer increased from T14 and reached the highest titers at T21 (1:10,240), but a slight decrease was observed from T28 (1:5120) to T56 (1:2560). Interestingly, antibodies detected with an SN test showed an analogous kinetic, but with slightly lower values (1:640). IgA antibodies in nasal secretion were detected at T28 (1:20), they increased at T35 (1:320) but rapidly decreased at T56 (1:40) (Figure 6).

**Figure 7:**
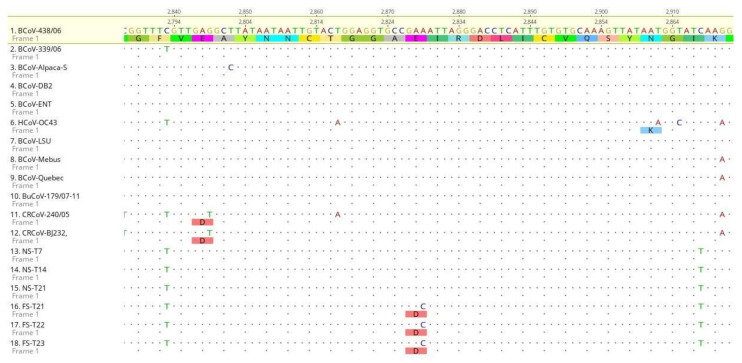
S gene sequence analysis. Alignment of partial nucleotide sequences and translated amino acid sequences in the spike protein of 18 *Betacoronavirus* strains: 12 reference strains (seq 1 to 12) (GenBank accession numbers EU14648.1, EF445634.1, DQ915164.2, DQ811784.2, AF391541.1, KX344031.1, AF058943.1, U00735.2, AF220295, EU19216.1, EU999954, KX432213.1) and 6 BCoV strains generated in this study (seq 13 to 18), from nasal and fecal swabs. BCoV strain 438/06 (GenBank accession number EU14648.1) was used as reference sequence for alignments. Specific amino acid substitution glu (E) to asp (D) among sequences, and the respective nt change A to C, is highlighted.

The sequence analysis of the BCoV S gene detected in the NS and in the FSs during the long shedding was sequenced and compared. Stool samples were collected at T22 and T23 to confirm sequence analysis from FS at T21. The 4.092 nt sequence of the S gene of NSs collected at T7, T14 and T21 and of the fecal samples collected at T21, T22 and T23 was translated in an expected 1.363aa and compared with the analogous sequences of the reference strains. By sequence comparison, an aa substitution (glu → asp) in position 943 was highlighted in the fecal strains, due to a nt change A to C. E → D substitution is characteristic and permanent in the fecal strains, as confirmed by a sequence comparison between the nasal samples at T7, T14 and T21, with the FSs collected at T21, T22 and T23, and was not shared by the other compared reference strains (Figure 7).

## 4. Discussion

The present study describes the evolution of a BCoV natural infection in a calf persistently infected with BVDV. Assuming that the calf was infected immediately before its arrival at the Department of Veterinary Medicine, two days after arrival, the animal showed hyperthermia (39.8 °C), a depressed general condition and a cough. Hyperthermia was constantly observed during the first 4 weeks of infection, with peaks during pronounced excretion, unlike previous studies where fever was absent or transient and generally occurred in severe cases and during BRD [22,23]. The calf developed moderate and intermittent nasal discharge and diarrhea. The appetite was not markedly decreased except on days of severe hyperthermia, while depression was constantly observed. The calf shed the virus both via the respiratory tract and the enteric apparatus up to three weeks post the onset of the symptoms and as previously observed, nasal shedding preceded fecal ones [14]. The shedding period was almost in agreement with other studies that demonstrated BCoV in fecal/nasal samples for about 2–10 days post infection (dpi) or up to 19 dpi when a PCR was performed [22,24]. A more recent study demonstrated that experimentally infected calves excreted detectable viral RNA intermittently in feces through day 35 dpi and in nasal secretions through day 28 dpi, but at this stage, the potential transmission is most likely meaningless [14]. Exceptionally, after experimental infection, the virus was detected sporadically, until 1085 dpi using a nested PCR [15], which is generally considered more sensitive than an RT-qPCR, but more vulnerable for contamination [25].

In concurrence with other studies, viral RNA was detected in fecal samples later than in nasal swabs (about 1 week later) [14,15,26] and nasal discharge showed a higher viral load than feces. In previous experimental infection trials, Saif et al. [27] demonstrated that in calves experimentally inoculated intranasal, BCoV was first detected in the nasal discharge and then in the feces, but when calves were infected orally, fecal detection preceded nasal detection. These observations led to the conclusion that the infection route can affect the phases of infection of the respiratory and intestinal tracts. In the described clinical case, the infectious route is unknown, but the data support the hypothesis that when calves are naturally infected, inhalation is the most common infection route, while the fecal–oral route could be more common with indirect virus spread [25]. A plausible scenario for the belated transit of BCoV to the intestine is that, following preliminary, intense and marked replication in the upper respiratory tract, BCoV spread to the gastrointestinal mucosa following ingestion of high viral titers of the virus coated in mucous secretions that act as a protection of the labile and enveloped virus, thus resulting in intestinal replication and fecal shedding [22].

It should also be emphasized that the calf was PI with BVDV; therefore, the BCoV infection may have assumed different connotations due to immunodepression. In a recent study, the fecal excretion of BCoV was significantly lower (*p* < 0.05) in the co-infected (BCoV/BVDV) calves than in calves infected with BCoV only, supposing an interference of BVDV with BCoV replication, probably mediated by BVDV-induced interferon type 1 [28]. On the contrary, our study highlighted high replication of both BCoV and BVDV and, moreover, an increased RNA BVDV load was detected in all the samples tested during the first two weeks of observation, when BCoV replication was highest. We can suppose that BVDV may have modulated the BCoV infection, exacerbating some clinical manifestations (hyperthermia) and the long viral shedding, and may have favored the onset of the interesting viral RNA mutations detected in the fecal samples at T21. Therefore, our data, despite requiring further investigations being data generated from a single animal, highlight the plasticity of BCoV that could generate the onset of mutations. Interestingly, despite the calf’s state of P.I., the BCoV infection was also characterized by deep seroconversion as observed using an IF test. High antibody titers persisted up to T60, suggesting that when BCoV RNA is long lastingly excreted from nasal/fecal secretions, detectable BCoV antibodies can be observed for a long time, as observed for up to 3 years [9,15]. Finally, the aa substitution detected in the S gene underlines that the examination of the sequence shifts in positive selection sites of CoVs’ genome and can provide data for the identification of new epidemic strains, genotypes or recombinant *betacoronaviruses* [29]. Nevertheless, the sequence of a single gene cannot define the appearance of new genotypes and, as observed for human CoV OC43, only the complete sequence analysis of BCoV RNA will allow for the identification of possible recombination or evolutionary events.

## 5. Conclusions

Extensive studies on the dual tissue pathogenesis, on the long shedding and on the mutational events that characterize a BCoV infection might contribute to an increased knowledge of CoVs infections also in humans [30].

## Figures and Tables

**Table 1:** Clinical scoring system. The score from each symptom was added to give a daily clinical score.

Score *	Body Temperature °	Sensory	Diarrhea	Nasal Discharge	Cough
0	<38.5	Bright, alert	Absent	Absent	Absent
1	38.6–39.5	Mildly depressed	Pasty	Serous/mucous	Sporadic
2	39.6–40.0	Moderately depressed	Runny	Mucopurulent	More than one cough every 10 min
3	>40.1	Severely depressed	Watery	Purulent	Severe

* Score: 0: Absent; 1: Mild; 2: Moderate; 3: Severe; ° Body temperature: 38.5 °C = 0; 0.5 °C ↔ 1 °C = 1; 1.1 °C ↔ 1.5 °C = 2; >1.5 °C = 3.

**Table 2:** Evaluation of clinical score and RT-qPCR Ct values. High rectal temperature and depression showed the best association with nasal shedding. The highest overall clinical score (in bold) observed at T7 is correlated with the high viral RNA detection (in bold).

dpi	Fever	Depression	Anorexia	Nasal Discharge	Cough	Diarrhea	Score °	Ct
**T1**	2	1	0	0	1	0	4	34.0
**T7**	2	2	1	0	1	0	**6**	**22.69**
**T14**	1	1	1	0	0	0	3	36.28
**T21**	0	1	0	0	1	1	3	38.48
**T28**	2	2	0	0	1	0	5	33.35
**T35**	0	1	0	1	0	0	2	neg

° Overall clinical score.

## Data Availability

The data presented in this study are available on request from the corresponding author. Full genome sequence will be publicly available in the GenBank database (https://www.ncbi.nlm.nih.gov/genbank/).
